# Supplementary material for: Investigating the influence of physiologically relevant hydrostatic pressure on CHO cell batch culture
Source: Sci Rep. 2021 Jan 8;11:162. doi: 10.1038/s41598-020-80576-8 (PMC7794228; doi:10.1038/s41598-020-80576-8)
Supplement: Supplementary file 1 — Supplementary Information 1. [file 41598_2020_80576_MOESM1_ESM.zip › S1/FLEX2/13Jan 30mmHg SS1 is C/Jan 8 C.pdf]

Data File C:\Users\P...ChemStation\1\Data\TK_CHO_IgG 2019-01-14 15-24-10\002-2-ML Jan8 C.D
Sample Name: ML Jan8 C

===

Acq. Operator : SYSTEM Seq. Line : 2
Sample Operator : SYSTEM
Acq. Instrument : HPLC-0039 Location : 2
Injection Date : 1/14/2019 3:29:57 PM Inj : 1
Inj Volume : 100.000 µl
Acq. Method : C:\Users\Public\Documents\ChemStation\1\Data\TK_CHO_IgG 2019-01-14 15-24-10\CHO_IgG_LC.M
Last changed : 12/4/2018 1:38:41 PM by SYSTEM
Analysis Method : C:\Users\Public\Documents\ChemStation\1\Data\TK_CHO_IgG 2019-01-14 15-24-10\CHO_IgG_LC.M (Sequence Method)
Last changed : 1/14/2019 3:46:42 PM by SYSTEM
(modified after loading) (Current integration events modified)

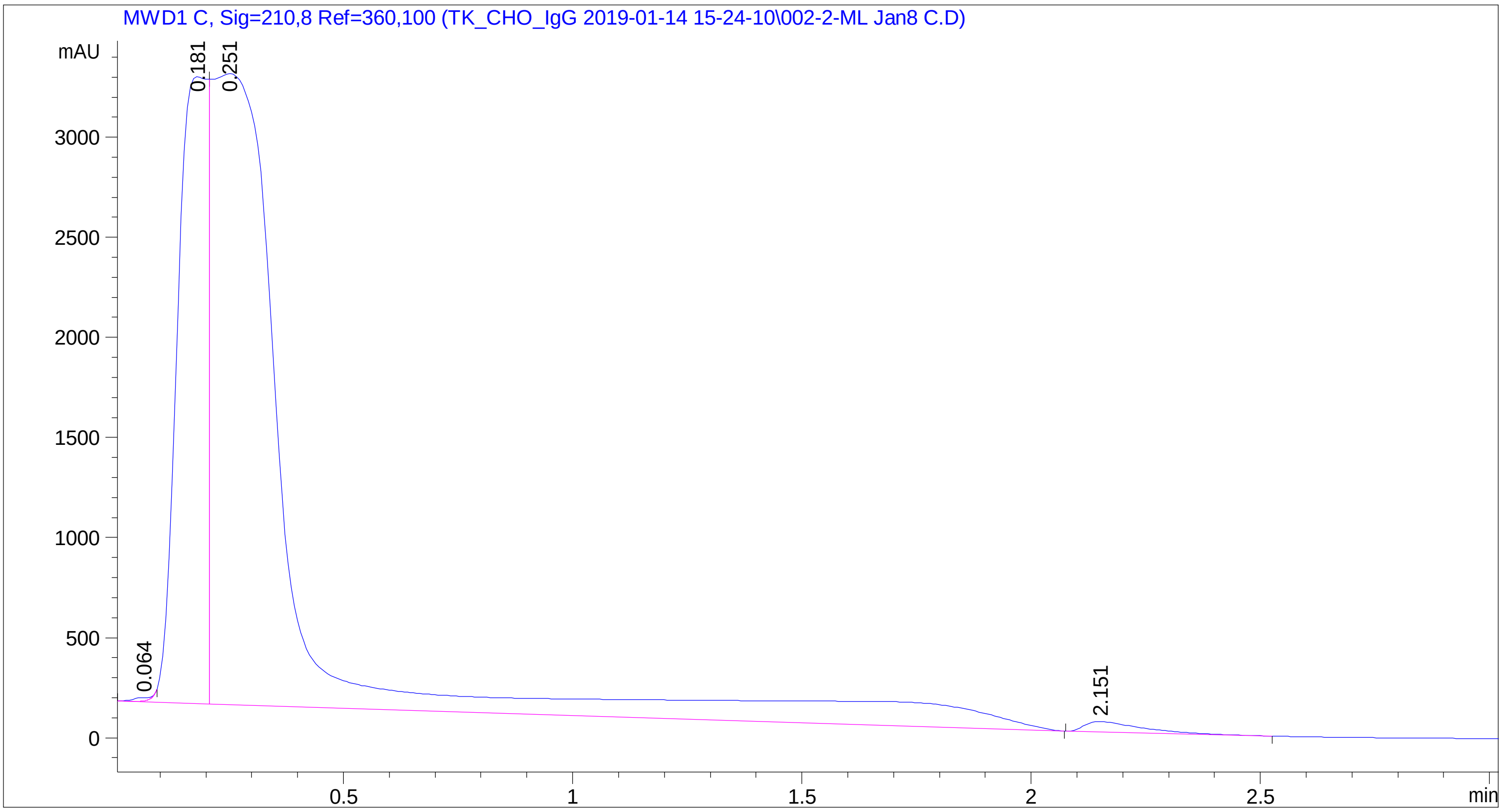

===

Area Percent Report

===

Sorted By : Signal
Multiplier : 1.0000
Dilution : 1.0000
Use Multiplier & Dilution Factor with ISTDs

Signal 1: MWD1 C, Sig=210,8 Ref=360,100

Peak #	RetTime [min]	Type	Width [min]	Area [mAU*s]	Height [mAU]	Area %
1	0.064	BV E	0.0369	42.39207	16.67370	0.0820
2	0.181	VV R	0.0686	1.41190e4	3131.08740	27.3208
3	0.251	VB	0.1692	3.70620e4	3150.52100	71.7163
4	2.151	BBA	0.1274	455.22275	52.39724	0.8809

Totals : 5.16786e4 6350.67934

===

*** End of Report ***
